# Photosynthesis in transition: the importance of wavelengths in the green gap for *Ocimum basilicum* L.

**DOI:** 10.3389/fpls.2026.1842244

**Published:** 2026-06-19

**Authors:** Luca Jokic, Isabell Pappert, Tran Quoc Khanh, Ralf Kaldenhoff

**Affiliations:** 1 Department of Applied Plant Sciences, Faculty of Biology, Technical University Darmstadt, Darmstadt, Germany; 2 Department of Adaptive Lighting Systems and Visual Processing, Faculty of Electrical Engineering and Information Technology, Technical University Darmstadt, Darmstadt, Germany

**Keywords:** action spectrum, basil, chlorophyll fluorescence, gas exchange, high irradiance, green light

## Abstract

In recent decades, the absorption spectrum as well as McCree’s action spectrum (350–750 nm) have served as the primary basis for photosynthesis research. Based on these spectra, it was long assumed that blue and red light may be used more efficiently for photosynthesis than green light. However, recent studies have demonstrated that green light is more effective than previously assumed, particularly at high irradiance conditions. Nevertheless, most studies still rely on the three standard wavelengths: 450, 527, and 660 nm. In this study, the effects of additional wavelengths on photosynthesis at irradiances of up to 5000 µmol m^-^² s^-^¹ were investigated in *Ocimum basilicum* L. using chlorophyll fluorescence analysis as well as gas exchange measurements. Following preliminary experiments that revealed variations in photosynthetic performance with plant age, as well as between leaf layers and the adaxial and abaxial leaf surfaces, a standardized measurement protocol was established and subsequently applied to evaluate the photosynthetic responses to different wavelengths. These wavelengths included deep blue (430 nm), cyan (485 nm), mint green (500 nm), orange (590 nm), and orange-red (625 nm). Unexpectedly, wavelengths within the “green gap” exhibited the highest CO_2_ assimilation rates, followed by deep blue and reddish wavelengths. Additionally, 4000 K neutral white light was supplemented with varying proportions of these wavelengths to determine the optimal white-light composition for photosynthesis. The most favorable results were obtained when white light was supplemented with 25% shorter wavelengths (430–527 nm).

## 1 Introduction

Photosynthesis forms the basis of nearly all life on Earth. Photoautotrophic organisms use solar energy to fix carbon dioxide, thereby providing the energy foundation for almost all living systems. The oxygen released during this process enables the existence of the flora and fauna as we know them today (Govindjee and Krogmann, 2004). Consequently, almost all life ultimately depends on light. The importance of light as a key factor in photosynthesis was recognized early in scientific research. In 1779, Jan Ingenhousz demonstrated that sunlight is responsible for oxygen production (Ingenhousz, 1779). Since then, significant advances have been made in photosynthesis research, particularly regarding the role of light. In this context, three major light-related concepts have become central to photosynthesis research.

The extended photosynthetic active radiation (ePAR) describes the spectral available for photosynthesis. It extends from 400 nm (visible violet light) to 750 nm (far red light) (Zhen et al., 2021).

However, plants cannot utilize all wavelengths within the photosynthetically active radiation (PAR) equally efficiently. The absorption spectrum is determined by the pigment composition of the photosystems and reflects which wavelengths are effectively absorbed in order to drive photosynthesis. These photosystems primarily consist of chlorophylls a and b and carotenoids, each of which exhibits distinct absorption maxima. Both chlorophyll a and chlorophyll b show pronounced absorption peaks in the blue and red regions (approximately 430–450 nm and 640–660 nm), while absorption in the green region is relatively low (Lichtenthaler and Buschmann, 2001). Carotenoids extend the absorption spectrum into the green wavelength range. Consequently, plants largely reflect or transmit green light due to the comparatively low absorption in this range, which is why plants appear green (Taiz et al., 2015). Historically this observation led to the assumption that blue and red wavelengths are most important for photosynthesis.

Established by McCree in 1971, the action spectrum builds upon the two previously described spectra. It describes the relative efficiency of photosynthesis across wavelengths. This spectrum has had a profound impact on research and indicates that blue light is used more efficiently than green light, although less efficiently than red light. Overall, the pattern broadly resembles that of the absorption spectrum. However, this representation is somewhat simplified McCree (1971) conducted his study using 22 plant species and analyzed the PAR in 20 nm increments. To achieve this, he used a broadband light source (metal halide lamp) in combination with a monochromator to generate specific wavelengths. A major limitation of this approach is the substantially reduced irradiance. As a consequence, maximum irradiances of only approximately 200 µmol m^-^² s^-^¹ could be achieved. In contrast, under natural outdoor conditions, plants are often exposed to irradiances of 2000–2500 µmol m^-^² s^-^¹ (Varella et al., 2011).

Furthermore, around 85% of blue and red light is absorbed within the upper 20% of the leaf, whereas green light penetrates deeper into the leaf tissue due to its lower absorption (Cui et al., 1991). This phenomenon is also reflected in carbon dioxide (CO_2_) fixation, as most CO_2_ assimilation driven by green light occurs in the middle and lower cell layers of the leaf (Sun et al., 1998). These findings are excellent examples of the sieve and detour effects, in which increased light penetration and internal reflection within the leaf allow green light to illuminate the leaf more uniformly, thereby promoting a more uniform distribution of photosynthetic activity across leaf tissue (Terashima et al., 2009). The combined effects of deeper penetration and more even internal light distribution contribute substantially to enhanced photosynthetic performance, particularly under high irradiance conditions. Several studies have already demonstrated that green light can be used more efficiently than red or blue light for CO_2_ assimilation in species such as lettuce (Liu and van Iersel, 2021), tomato (Lanoue et al., 2017), common chicory (Landi et al., 2020), maize (Pappert et al., 2025), and basil (Jokic et al., 2025) when irradiance approaches or exceeds the light saturation point. Another advantage of the lower absorption of green light lies in its thermal properties. Compared to blue and red light, green light causes less heating of the leaf, thereby reducing the risk of thermal stress and potential oxidative damage, which can occur under intense blue or red illumination (Jokic et al., 2025; Pappert et al., 2025).

Taking these factors into consideration, it remains unclear whether McCree’s spectrum is fully representative under high irradiance conditions. Furthermore, questions arise regarding the wavelengths within the so-called “green gap” (480–580 nm), which have received relatively little attention in modern research. However, recent advances in lighting technology, particularly the development of light-emitting diode (LED) technology, have enabled us to overcome many of the limitations of previous studies. This enables further investigation of wavelength-dependent photosynthesis and addresses said questions. The objective of this study was to investigate whether wavelengths within the “green gap” contribute more strongly to photosynthetic performance than conventionally assumed and may therefore provide new perspectives for photosynthesis research and the optimization of plant cultivation.

## 2 Materials and methods

### 2.1 Plant material and growth conditions

*Ocimum basilicum* L. cv. Genoveser (basil) was cultivated in a greenhouse in Darmstadt, Germany. Environmental parameters within the greenhouse were strictly controlled to minimize external influences. Average day and night temperatures of 21.5 °C were maintained, alongside ambient atmospheric CO_2_ concentrations and a relative humidity of 80%. Plants were grown under a 12-hour photoperiod. Illumination was provided by a PQ5W S2.1 Gen2 LED system (SANlight GmbH, Schruns, Austria) with a white-to-red ratio of 3:1, a color temperature of 3500 K and an average irradiance of 250 µmol m^-^² s^-^¹. The seeds were purchased from Kiepenkerl (Bruno Nebelung GmbH, Everswinkel, Germany). They were sown in Fruhstorfer potting substrate type LD 80 (Hawita Gruppe GmbH, Vechta, Germany), according to the manufacturer’s instructions. The substrate is characterized by a fine structure, a pH value of 5.8, a salinity of 1.1 g L^-^¹, and contains a starter fertilization of 1.0 kg m^-3^ and 3.0 kg m^-^³ of coated controlled-release fertilizer. No additional fertilizer was applied during cultivation. Plants were irrigated every second day using a water bath system. Pots were placed into the water bath until the substrate was visibly saturated, after which they were removed and allowed to drain. The seeds germinated three days after sowing and the seedlings were transplanted one week later. After an additional week, the plants were repotted from 7 cm diameter pots to 12 cm diameter pots. Three weeks after sowing, the basil plants had developed leaves larger than 10 cm², which met the requirements of the respective measurement methods. The plants were examined throughout a growth period of up to seven weeks after sowing. By the seventh week, basil plants had formed their first flower clusters.

### 2.2 Study site and experimental design

All measurements were conducted under controlled laboratory conditions in Darmstadt, Germany. A series of six interrelated experiments formed the experimental design. During the initial experimental phase, factors potentially affecting the quantification of photosynthesis were identified. These included the effects of applying either a gradual light curve or direct exposure to defined irradiance levels, as well as the influence of plant age, leaf layer and measurement configuration on photosynthetic responses. Based on these initial investigations, a standardized measurement protocol was established, including the use of plants within a 3–6 week age range, the application of a light curve protocol, and measurements restricted to a specific leaf layer and side. In each experiment, one of the youngest fully developed upper leaf pairs was measured, except in 3.3, where leaves from the upper, middle, or lower canopy levels were examined. Using this standardized protocol, the effects of monochromatic and spectral supplementation of white light at different irradiance levels on photosynthetic performance were subsequently analyzed. All measurements were taken between 08:00 and 22:00 CET. Ten individuals were used per treatment condition in each experiment, resulting in a total of 400 plants being measured.

### 2.3 Parameter measurement and methods

Two complementary measurement systems were used to quantify photosynthesis. Chlorophyll fluorescence measurements were performed to analyze light-dependent energy conversion, whereas gas exchange measurements were conducted to quantify CO_2_ assimilation.

#### 2.3.1 Gas exchange analysis

Prior to the measurements, the plants were dark-adapted overnight. To calibrate the measuring system and record the plant’s initial physiological state, a baseline measurement of 10 minutes was conducted in darkness prior to each light treatment. These measurements served as a dark reference state, in which no photosynthetic CO_2_ assimilation occurred and only respiratory metabolism was active. Initial adaptation to the first irradiance level required 45 minutes, whereas subsequent irradiance increases required 20 minutes of acclimation. Consequently, each gas exchange analysis took a minimum of 55 minutes for one irradiance level and up to 135 minutes for four additional irradiance levels. The CO_2_ assimilation rate (A) in µmol m^-^² s^-^¹, stomatal conductance [GH_2_O] in µmol m^-^² s^-^¹, transpiration rate (E) in mmol m^-^² s^-^¹ and leaf temperature (T) in °C were quantified using a GFS-3000 gas exchange system (Heinz Walz GmbH, Effeltrich, Germany). Measurements were recorded at five-second intervals under controlled conditions with a CO_2_ concentration of 400 ppm, a relative humidity value of 57%, a chamber temperature of 25 °C, a corresponding vapor pressure deficit of 1.4 kPa and an airflow rate of 750 µmol s^-^¹. The measured leaf area covered 3 cm² of the chamber window. Data analysis was based on the mean value of the final 90 seconds of each irradiance level, after the plants had reached a steady-state following full adaptation to the respective light treatment.

#### 2.3.2 Chlorophyll fluorescence analysis

**Figure 1:**
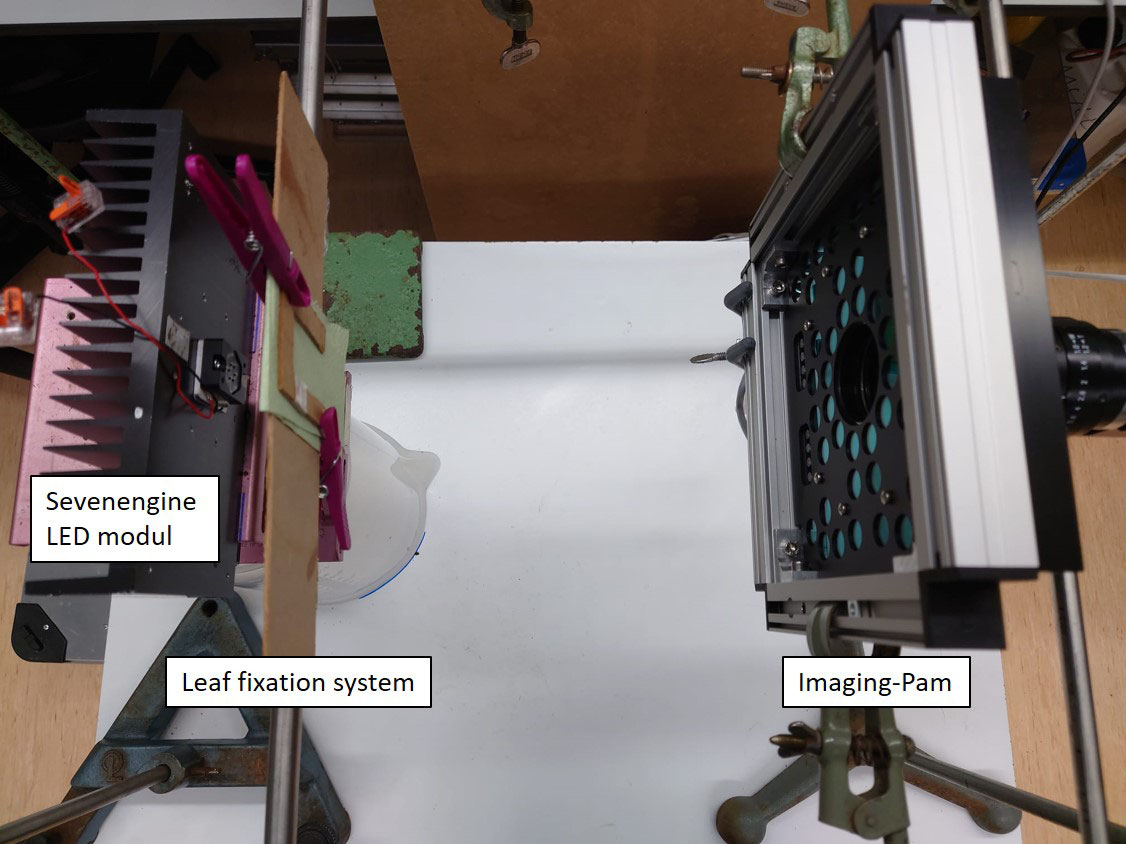
Image of the experimental setup of the Imaging-Pam.

Prior to measurement, the plants were dark-adapted overnight. A baseline measurement to record the plant’s initial physiological state took place in the beginning of each experiment. As pulse-amplitude-modulated chlorophyll fluorescence measurements use short saturating light pulses, this initial step required only a few seconds. Initial adaptation to the first irradiance level required 15 minutes, whereas subsequent irradiance increases required 8 minutes of acclimation. Consequently, each chlorophyll fluorescence analysis took a minimum of 22 minutes for one light irradiance level and up to 87 minutes for nine additional irradiance levels. An Imaging-PAM M as well as a Junior-PAM (Heinz Walz GmbH, Effeltrich, Germany) were used to obtain additional parameters, including the quantum yield of photosystem II [Y(II)] and non-photochemical quenching [NPQ]. Due to the size of the Imaging-PAM and the proximity of the external light source to the illuminated plant, the camera had to be removed from its original mounting and integrated into a modified experimental setup. In this configuration, the Imaging-PAM camera and the external light source were positioned opposite each other along a horizontal axis. For each measurement, a leaf was carefully clamped into a centrally mounted holder (**Figure 1**). Subsequently, either the abaxial side of the leaf was illuminated while fluorescence was measured on the adaxial side, or vice versa. Each irradiance level was evaluated using the mean value of the final 90 seconds after the plant had reached a steady state following full adaptation.

#### 2.3.3 Light source

**Figure 2:**
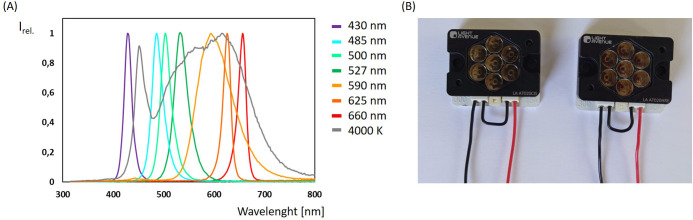
**(A)** Emission spectra (relative intensity versus wavelength in nm) of Sevenengine LED modules from Chips 4 Light GmbH; 430 nm (deep blue), 485 nm (cyan), 500 nm (mint green), 527 nm (green), 590 nm (orange), 625 nm (orange-red), 660 nm (red) and 4000 K (neutral white) and **(B)** Image of the used Sevenengine LED modules.

Seven engine LED modules (Chips 4 Light GmbH in Sinzing, Germany) were used as the external light source for the experiments (**Figure 2B**). These high-performance LED modules have a 20-degree beam angle, enabling uniform and targeted illumination. Advanced thermal management and a broad spectral range allowed irradiances of up to 10,000 µmol m^-^² s^-^¹, covering wavelengths ranging from 367 nm to 940 nm for quasi-monochromatic radiation and 2600 K to 6000 K for polychromatic white LED light. Emission spectra of these modules were measured using a SpectraPen Mini (Photon Systems Instruments (PSI), **Figure 2A**). Irradiances were measured using a ULM-500 light meter and data logger equipped with a cosine-corrected mini quantum sensor (MQS-B) and a spherical micro quantum sensor (US-SQS/L, Heinz Walz GmbH, Effeltrich, Germany).

### 2.4 Statistical analysis and visual representation

To assess statistical differences between plant groups, a one-way analysis of variance (ANOVA) was performed with a significance level of p ≤ 0.05. When significant effects were detected, a *post-hoc* test (Tukey’s HSD) was applied to identify pairwise differences between groups. All figures and tables present mean values ± standard deviation of the respective plant groups. The light curves were generated by plotting the measured values against increasing irradiance levels.

## 3 Results

### 3.1 Unexpected stability of photosynthetic performance under extreme irradiance

**Figure 3:**
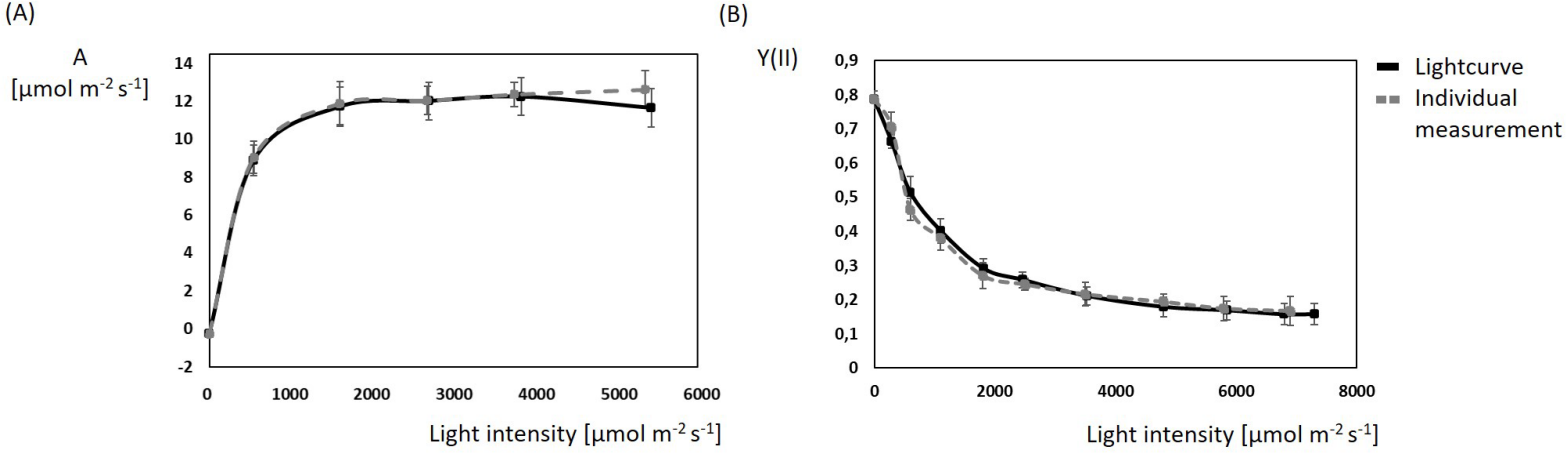
Comparison of **(A)** the CO_2_ assimilation rate [A] in µmol m^-^² s^-^¹ and **(B)** the quantum yield of photosystem II [Y(II)] as a function of irradiance in µmol m^-^² s^-^¹ (4000 K for A and 2600 K for B), obtained through a light curve measurement procedure and individual measurements (n = 10 per light curve and per irradiance of individual measurements).

Due to the specific design of the Sevenengine LED modules, irradiance of up to 10,000 µmol m^-^² s^-^¹ can be applied to a precisely targeted area, corresponding to approximately four to five times natural solar irradiance. Two different approaches were employed to expose the leaves to such extreme irradiance levels. In the first approach, the principle of natural sunrise was simulated using a light curve, allowing the plants to gradually adapt to irradiances exceeding those of natural sunlight. Alternatively, the plants were directly exposed to these extreme irradiance levels (individual measurements). Based on these two experimental approaches, the hypothesis was formulated that direct exposure without gradual adaptation would overload the leaf, resulting in minimal or no photosynthetic activity. This hypothesis was tested using ten individuals for each light curve and for each individual irradiance treatment (**Figure 3**).

No significant difference in CO_2_ assimilation could be observed up to an irradiance of 5000 µmol m^-^² s^-^¹, nor could any difference in the quantum yield of PS II be observed between a light curve and an individual measurement up to an irradiance of 7000 µmol m^-^² s^-^¹. Maximum CO_2_ assimilation was reached at 1500 µmol m^-^² s^-^¹ and remained around 12 µmol m^-^² s^-^¹ up to 5000 µmol m^-^² s^-^¹ (**Figure 3A**; **Supplementary Table 1**). Quantum yield initially started at a value of 0.79 for both the light curve and the individual measurements, following a negative exponential curve which levelled off at 0.16 at 7500 µmol m^-^² s^-^¹ (**Figure 3B**; **Supplementary Table 2**). Accordingly, the hypothesis could be rejected. Direct exposure to extreme intensities did not impair photosynthetic performance in basil.

### 3.2 Progressive decoupling of stomatal conductance and CO_2_ assimilation during plant development

**Figure 4:**
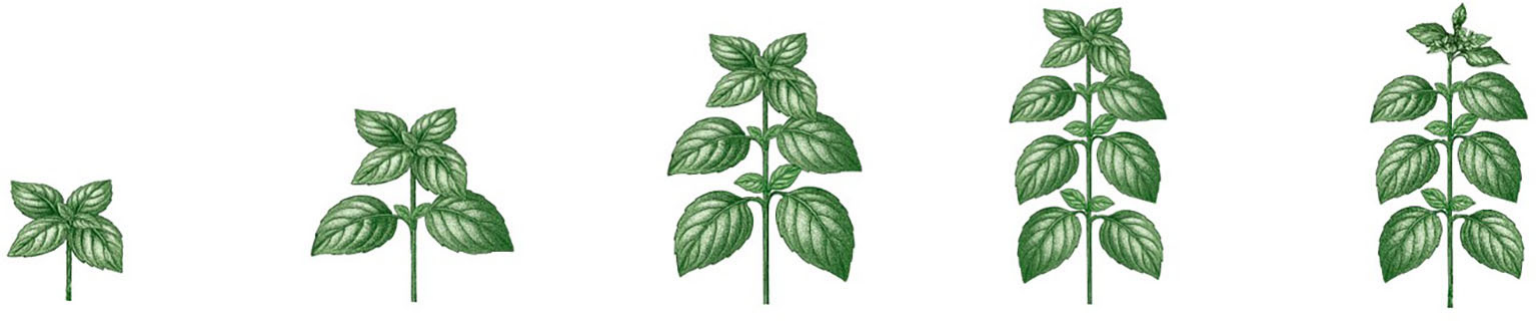
Schematic representation of a seven-week growth period of a basil plant (*Ocimum basilicum* L.), beginning in the third week.

To investigate how photosynthetic performance varies with plant age, ten plants from each age group were measured throughout the seven-week growing season, as depicted in **Figure 4**. Measurements began in the third week (**Table 1**).

**Table 1:** Comparison of CO_2_ assimilation rate in µmol m^-^² s^-^¹, stomatal conductance in µmol m^-^² s^-^¹, transpiration rate in mmol m^-^² s^-^¹, leaf temperature in °C, quantum yield of PS II and non-photochemical quenching at a irradiance of 1500 µmol m^-^² s^-^¹ (4000 K) for gas exchange measurements or 2500 m^-^² s^-^¹ for chlorophyll fluorescence measurements for different age groups (n = 10 per age group).

Parameters/age of plant	3 weeks	4 weeks	5 weeks	6 weeks	7 weeks
CO_2_ assimilation rate [µmol m^-^² s^-^¹]	12.34 ± 0.50 ^a^	11.86 ± 0.81 ^a^	12.24 ± 1.08 ^a^	12.23 ± 0.94 ^a^	9.80 ± 0.88 ^b^
Stomatal conductance [µmol m^-^² s^-^¹]	244.41 ± 12.88 ^a^	220.28 ± 23.17 a^,b^	171.50 ± 20.47 ^c,d^	183.08 ± 19.49 ^c,b^	140.57 ±19.29 ^d^
Transpiration rate [mmol m^-^² s^-^¹]	2.92 ± 0.11 ^a^	2.55 ± 0.16 ^b^	2.37 ± 0.26 ^b,c^	2.39 ± 0.25 ^b,c^	1.95 ± 0.23 ^c^
Leaf temperature [°C]	24.77 ± 0.08 ^a^	24.97 ± 0.12 a^,b^	25.01 ± 0.05 ^b^	25.01 ± 0.04 ^b^	25.28 ± 0.17 ^c^
Quantum yield of PS II	0.25 ± 0.02 ^a^	0.24 ± 0.02 ^a^	0.25 ± 0.02 ^a^	0.23 ± 0.02 ^a^	0.24 ± 0.03 ^a^
Non-photochemical quenching	0.51 ± 0.02 ^a^	0.54 ± 0.02 ^a^	0.52 ± 0.04 ^a^	0.52 ± 0.02 ^a^	0.52 ± 0.03 ^a^

Statistical significance was determined by one-way ANOVA (p ≤ 0.05). Different lowercase letters indicate statistically significant differences between treatments.

CO_2_ assimilation rates did not differ between age groups of three to six weeks. At an irradiance of 1500 µmol m^-^² s^-^¹, these rates ranged from 11.86 to 12.34 µmol m^-^² s^-^¹. However, seven-week-old plants had a significantly lower maximum CO_2_ assimilation rate of 9.8 µmol m^-^² s^-^¹ than younger plants. Stomatal conductance and transpiration rates decreased with increasing age. Leaf temperature changed only slightly at an irradiance of 1500 µmol m^-^² s^-^¹. (**Table 1**). Due to the plants’ nearly identical CO_2_ assimilation up to six weeks of age and the associated lower gas exchange, resulting from decreasing stomatal conductance with increasing age, photosynthetic efficiency increased up to this age. However, from the first flower formation in the seventh week, this increase in photosynthetic efficiency declined again. This contrasts with the results of the chlorophyll fluorescence analysis. No significant decrease in quantum yield or increase in non-photochemical quenching was observed between the age groups. Quantum yield varied around 0.24, while non-photochemical quenching ranged from 0.51 to 0.54 (**Table 1**).

### 3.3 Intra-plant vertical gradients in photosynthetic capacity

By four to five weeks of age, basil plants had developed up to three leaf layers (**Figure 4**), with the lowest layer containing the oldest leaves. Their different photosynthetic performance represented another physiological parameter analyzed in this study (**Table 2**).

**Table 2:** Comparison of CO_2_ assimilation rate in µmol m^-^² s^-^¹, stomatal conductance in µmol m^-^² s^-^¹, transpiration rate in mmol m^-^² s^-^¹, leaf temperature in °C, quantum yield of PS II and non-photochemical quenching at an irradiance of 1500 µmol m^-^² s^-^¹ (4000 K) for gas exchange measurements or 2500 m^-^² s^-^¹ for chlorophyll fluorescence measurements for different leaf layers (n = 10 per leaf layer).

Parameters/leaf level	Upper leaves	Middle leaves	Lower leaves
CO_2_ assimilation rate [µmol m^-^² s^-^¹]	11.83 ± 0.94 ^a^	8.82 ± 0.76 ^b^	5.10 ± 0.51 ^c^
Stomatal conductance [µmol m^-^² s^-^¹]	193.77 ± 19.96 ^a^	112.47 ± 12.16 ^b^	96.19 ± 8.16 ^b^
Transpiration rate [µmol m^-^² s^-^¹]	2.46 ± 0.21 ^a^	1.65 ± 0.13 ^b^	1.38 ± 0.10 ^c^
Leaf temperature [°C]	25.00 ± 0.09 ^a^	0.26 ± 0.01 ^a^	0.25 ± 0.01 ^a^
Quantum yield of PS II	0.25 ± 0.02 ^a^	0.26 ± 0.01 ^a^	0.25 ± 0.01 ^a^
Non-photochemical quenching	0.52 ± 0.03 ^a^	0.53 ± 0.04 ^a^	0.52 ± 0.03 ^a^

Statistical significance was determined by one-way ANOVA (p ≤ 0.05). Different lowercase letters indicate statistically significant differences between treatments.

As the distance between the leaf layer and the light source increased, distinct decreases occurred in CO_2_ assimilation, transpiration, and stomatal conductance (**Table 2**). The reduction in stomatal opening in the lower leaves led to less transpiration, as well as less transpiration-related cooling. Therefore, unlike the previous three parameters, the uppermost leaf layer had the lowest temperature, while a significant temperature increase was observed in the middle and lower leaf layers. Contrary to expectations, no significant differences were observed in the efficiency of converting light energy into chemical energy or dissipating excess energy in the form of heat among the leaf layers. As in the previous experiment, the quantum yield was approximately 0.25, and the non-photochemical quenching yield fluctuated around 0.52 (**Table 2**).

### 3.4 Leaf-side-dependent differences in chlorophyll fluorescence

**Figure 5:**
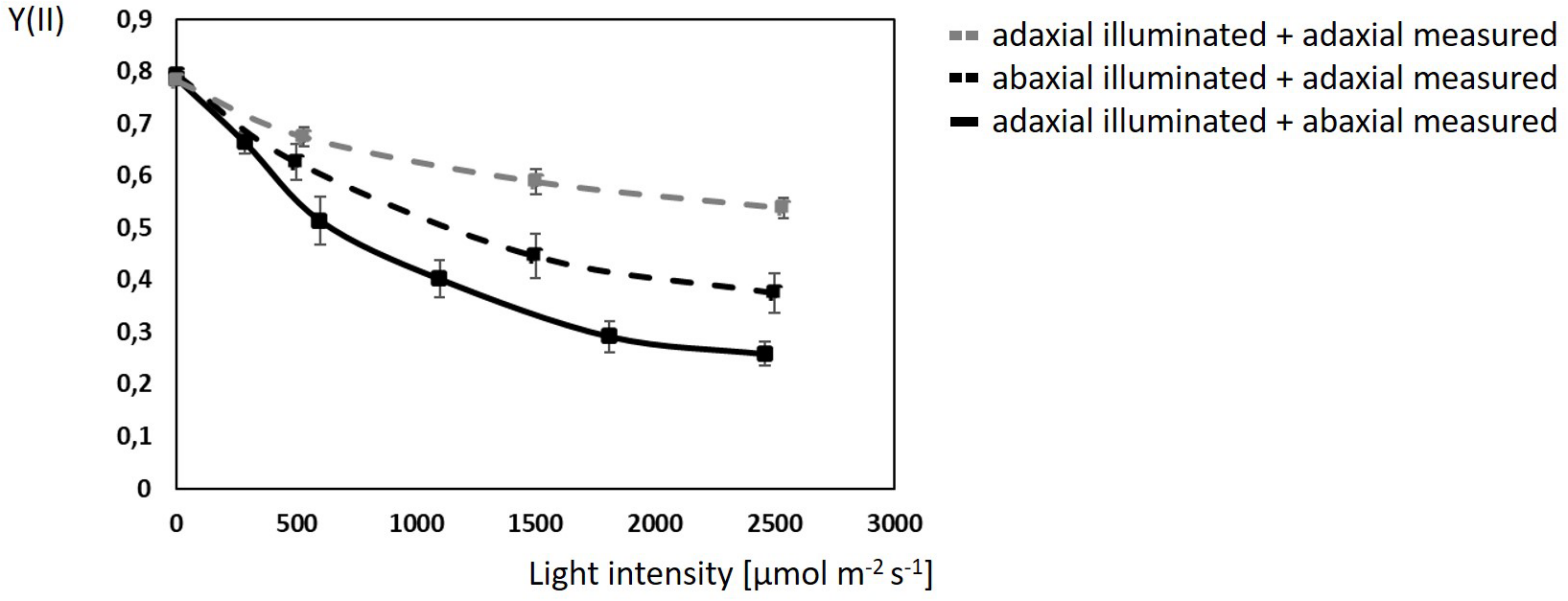
Comparison of the quantum yield of PS II [Y(II)] as a function of irradiance in µmol m^-^² s^-^¹ between the different experimental approaches to measure chlorophyll fluorescence (n = 10 per measurement condition).

The horizontal Imaging-PAM setup and the vertical Junior-PAM setup resulted in three options for illuminating and measuring a single leaf. With the Imaging-PAM in the horizontal setup, the upper side of the leaf (adaxial) was illuminated while the underside (abaxial) was measured, or vice versa. The vertical setup of the Junior-PAM, on the other hand, illuminated and measured the upper side of the leaf (**Figure 5**).

In all scenarios, measurements began in the dark state with a quantum yield of 0.79 and proceeded slightly negatively exponentially. From an intensity of 500 µmol m^-^² s^-^¹ onwards, the measurement scenarios could be clearly distinguished. At an irradiance of 2500 µmol m^-^² s^-^¹, the quantum yield ranged from 0.26 to 0.54, with an intermediate value of 0.38 depending on whether the upper or lower leaf surface was illuminated and measured. The measurement variant in which the upper side of the leaf was both illuminated and measured resulted in the highest quantum yield. In contrast, the combination of illuminating the upper side and measuring the underside achieved the lowest quantum yield (**Figure 5**; **Supplementary Table 3**). A mirrored response pattern with an identical differentiation of the three measurement conditions was also observed for NPQ (**Supplementary Figure 1**). Therefore, direct comparison between the three measurement variants is impossible because leaf-side-specific chlorophyll fluorescence was detected.

### 3.5 Beyond the conventional spectrum: photosynthesis across underexplored wavelengths

**Figure 6:**
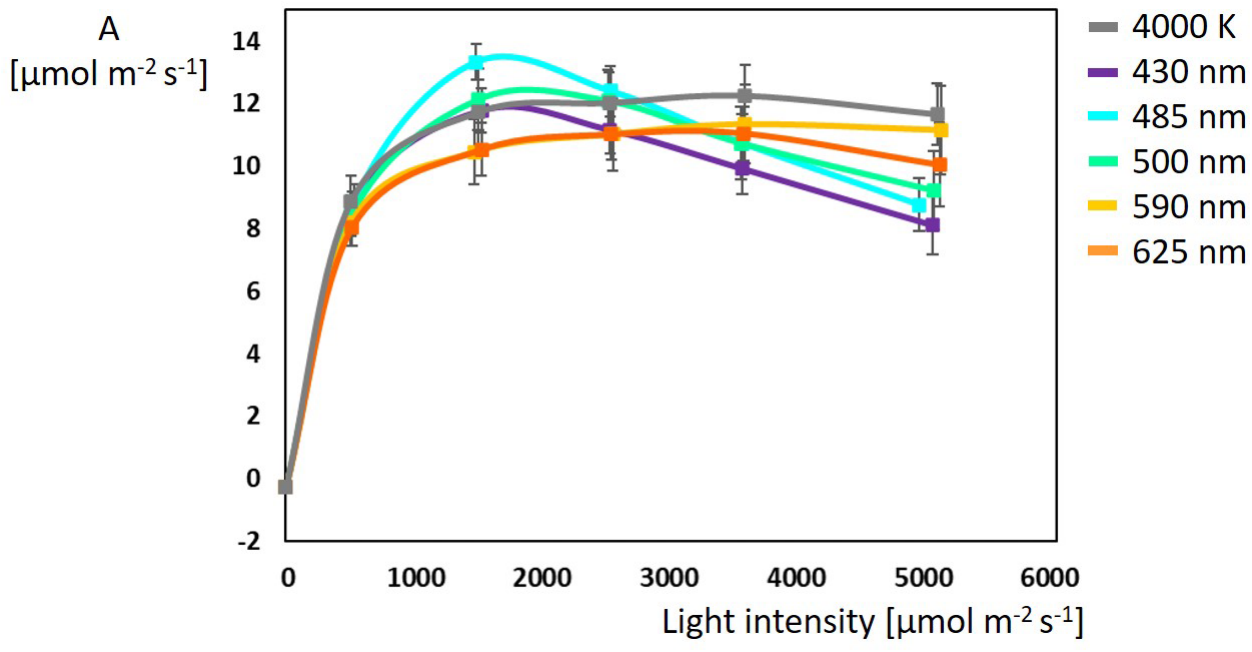
Comparison of the CO_2_ assimilation rate [A] in µmol m^-^² s^-^¹ as a function of irradiance in µmol m^-^² s^-^¹, under different light colors (neutral white: 4000 K, deep blue: 430 nm, cyan: 485 nm, mint green: 500 nm, orange: 590 nm, and orange-red: 625 nm, n = 10 per light color).

Due to the exceptionally broad spectral coverage of Sevenengine LED modules, the influence of rarely studied wavelengths on photosynthesis was analyzed (**Figure 6**).

Under dark conditions, CO_2_ assimilation was initially measured at -0.27 µmol m^-^² s^-^¹ and increased significantly with increasing irradiance until reaching its saturation point at 1500 µmol m^-^² s^-^¹. Beyond the light saturation point, a plateau formed up to 5000 µmol m^-^² s^-^¹ with neutral white light at 4000 K and orange light (590 nm), and up to 3500 µmol m^-^² s^-^¹ with orange-red light (625 nm). In the deep blue to mint green wavelength range (430, 485, and 500 nm), however, a pronounced CO_2_ assimilation maximum was observed at 1500 µmol m^-^² s^-^¹. As irradiance increased, the CO_2_ assimilation rate decreased distinctively due to photoinhibitory effects. Cyan light (485 nm) achieved the highest CO_2_ assimilation rate of 13.33 µmol m^-^² s^-^¹ at 1500 µmol m^-^² s^-^¹, significantly differing from every other tested wavelength except 500 nm (**Table 3**). Neutral white, mint green, and deep blue light reached a maximum of 11.73 to 12.13 µmol m^-^² s^-^¹, while red wavelengths maximum CO_2_ assimilation rates were considerably lower at approx. 10.5 µmol m^-^² s^-^¹ (**Figure 6**).

**Table 3:** Comparison of CO_2_ assimilation rate in µmol m^-^² s^-^¹ at an irradiance of 1500 µmol m^-^² s^-^¹ (neutral white: 4000 K, deep blue: 430 nm, cyan: 485 nm, mint green: 500 nm, orange: 590 nm, and orange-red: 625 nm, n = 10 per light color) depicted from **Figure 6**.

Wavelength	CO_2_ assimilation rate [µmol m^-^² s^-^¹] at 1500 µmol m^-^² s^-^¹
Cyan (485 nm)	13.33 ± 0.57 ^a^
Mint green (500 nm)	12.13 ± 0.99 ^a,b^
Deep blue (430 nm)	11.79 ± 0.71 ^b,c^
Neutral white (4000 K)	11.73 ± 1.03 ^c,b^
Orange-red (625 nm)	10.54 ± 0.84 ^c^
Orange (590 nm)	10.46 ± 1.03 ^c^

Statistical significance was determined by one-way ANOVA (p ≤ 0.05). Different lowercase letters indicate statistically significant differences between treatments.

**Figure 7:**
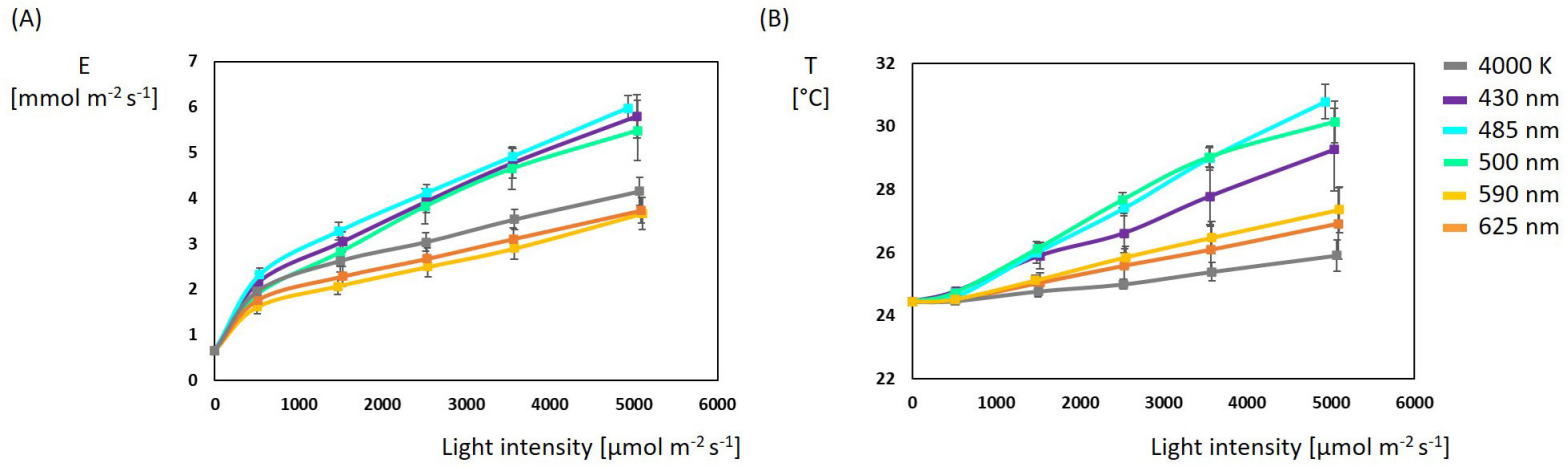
Comparison of **(A)** the transpiration rate [E] in mmol m^-^² s^-^¹ and **(B)** the leaf temperature [T] in °C as a function of irradiance in µmol m^-^² s^-^¹, under different light colors (neutral white: 4000 K, deep blue: 430 nm, cyan: 485 nm, mint green: 500 nm, orange: 590 nm, and orange-red: 625 nm, n = 10 per light color).

Significant differences in transpiration rate were also observed between light treatments. At 5000 µmol m^-^² s^-^¹, the transpiration rate was highest for deep blue to mint green wavelengths, ranging from 5.48 to 5.98 mmol m^-^² s^-^¹. Neutral white light and the two red wavelengths induced notably lower values at 4.15 and 3.66/3.73 mmol m^-^² s^-^¹, respectively. However, this clear differentiation only occurred at an irradiance of 1500 µmol m^-^² s^-^¹ and increased with rising irradiance (**Figure 7A**; **Supplementary Table 4**). A similar pattern was observed for leaf temperature. Leaf temperature under dark conditions initially averaged 24.45 °C. When the respective light sources were activated, the leaf temperatures rose linearly. Cyan and mint green light produced the strongest increase, reaching temperatures over 30 °C at maximum irradiance. Deep blue light produced a slightly lower increase of 29.26 °C. The two reddish wavelengths caused a moderate increase of 26.92 to 27.36 °C. White light resulted in the lowest thermal load at 25.91 °C (**Figure 7B**; **Supplementary Table 5**).

### 3.6 Improving photosynthetic efficiency through spectral extension of white light

**Figure 8:**
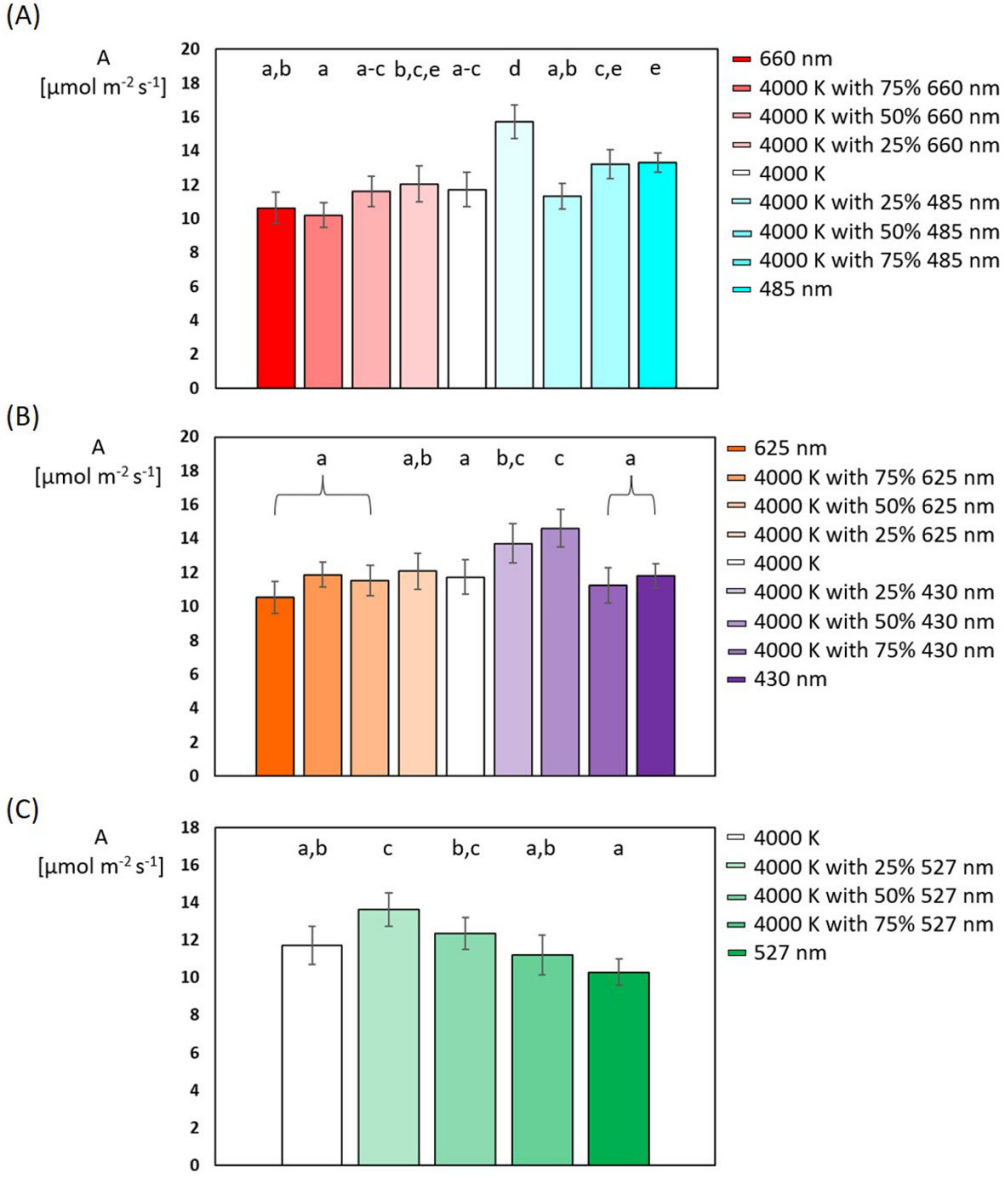
Comparison of CO_2_ assimilation rate [A] in µmol m^-^² s^-^¹ under different white light supplements: **(A)** neutral white: 4000 K with 0, 25, 50, 75, or 100% red: 660 nm or cyan: 485 nm light, **(B)** neutral white: 4000 K with 0, 25, 50, 75, or 100% orange-red: 625 nm or deep blue: 430 nm light and **(C)** neutral white: 4000 K with 0, 25, 50, 75, or 100% green: 527 nm light at an irradiance of 1500 µmol m^-^² s^-^¹ (n = 10 per light combination). Statistical significance was determined by one-way ANOVA (p ≤ 0.05). Different lowercase letters indicate statistically significant differences between treatments.

To investigate how white light can be supplemented to maximize CO_2_ assimilation in basil, we utilized the spectral diversity of the Sevenengine LED modules, precise irradiance control, and simultaneous control of two LED chips. Neutral white light with a color temperature of 4000 K was chosen for the base lighting. To supplement the white light, the following wavelengths were selected: 430 nm (deep blue), 485 nm (cyan), 527 nm (green), 625 nm (orange-red), and 660 nm (red). The effects of monochromatic green and red light at 527 nm and 660 nm, with an irradiance of up to 8000 µmol m^-^² s^-^¹, on photosynthesis can be seen in Jokic’s work from 2025 (Jokic et al., 2025). Photosynthesis was measured while varying the proportion of white-light admixture from 0% to 100% in 25% increments, while maintaining a constant total irradiance of 1500 µmol m^-^² s^-^¹. (**Figure 8**).

**Figure 8A** shows red light supplementation (660 nm) on the left, neutral white reference lighting (4000 K) in the middle, and cyan light supplementation (485 nm) on the right. Supplementation with red light (660 nm) did not lead to a significant increase in the CO_2_ assimilation rate. Instead, as the proportion of red light increased, there was a slight, though not significant, decrease to 10.65 µmol m^-^² s^-^¹. An opposite trend was observed with cyan light supplementation. A proportion of 25% cyan light significantly increased the CO_2_ assimilation rate from 11.73 µmol m^-^² s^-^¹ to 15.74 µmol m^-^² s^-^¹. However, as the proportion of cyan light increased further, the CO_2_ assimilation rate decreased again, approaching the initial value of monochromatic cyan light irradiation (13.33 µmol m^-^² s^-^¹). Supplementing with 25% cyan light slightly increased stomatal conductance and transpiration rate, while slightly raising the temperature (**Supplementary Figure 3**).

Supplementing white light with a different red-light wavelength (625 nm) produced similar results: no increase in CO_2_ assimilation was observed. However, supplementing with 25% or 50% deep blue light (430 nm) led to significant increases in CO_2_ assimilation, reaching 13.72 and 14.61 µmol m^-^² s^-^¹ respectively. As the blue light percentage increased, the CO_2_ assimilation rate decreased, approaching the rate of monochromatic light at 11.79 µmol m^-^² s^-^¹ (**Figure 8B**). The differences in stomatal conductance and transpiration rate were minimal under monochromatic and supplemented light irradiation. Leaf temperature increased gradually with increasing proportions of blue light (**Supplementary Figure 4**).

Adding 25% green light significantly increased the CO_2_ assimilation rate to 13.64 µmol m^-^² s^-^¹. Gradually increasing the proportion led to a decrease in CO_2_ assimilation, approaching the level of monochromatic green light (10.29 µmol m^-^² s^-^¹) (**Figure 8C**). Supplementing with green light did not lead to significant changes in transpiration rate, stomatal conductance, or leaf temperature (**Supplementary Figure 5**).

The results indicate that a shift in the white light spectrum toward warmer color temperatures was associated with lower photosynthetic efficiency. In contrast, a shift toward cooler white light spectra increased efficiency. However, there was a spectral optimum; a further shift toward cold white light negatively affected photosynthesis, leading to a decrease in the CO_2_ assimilation rate.

## 4 Discussion

### 4.1 Light tolerance of the photosynthetic apparatus under white light

Basil reached its light saturation point at an irradiance of 1500 µmol m^-^² s^-^¹. This value is consistent with those reported in similar studies. However, maximum CO_2_ assimilation rates reported in these studies were considerably higher than those observed in the present study. While one study reported maximum CO_2_ assimilation rates of 27.75 and 23.96 µmol m^-^² s^-^¹ for two varieties of basil (Coutinho et al., 2022), another observed a maximum rate of 23.9 µmol m^-^² s^-^¹ (Park et al., 2016). In contrast, this study measured a maximum CO_2_ assimilation rate of 11.7 µmol m^-^² s^-^¹ under neutral white light (4000 K), which is less than half of the rates reported in previous studies. A further comparison can be drawn to the results reported by (Jokic et al., 2025) who investigated basil photosynthesis under warm white light (2600 K). In that study, maximum CO_2_ assimilation rates amounted to less than one-third of the values reported by (Park et al., 2016; Coutinho et al., 2022). The considerable differences in absolute CO_2_ assimilation rates among these studies are likely attributable to several factors. First, different basil varieties were used, which may differ in their photosynthetic capacity. Second, the spectral composition of the measurement light was not specified in the previous studies. White light covers a color temperature range from 1500 to 8000 Kelvin. The influence of light quality is evident when comparing the results of the present study, conducted using neutral white light (4000 K), with the results reported with warm white light (2600 K) under identical experimental conditions (Jokic et al., 2025). Differences in spectral composition, particularly in the proportion of blue and red wavelengths, are known to affect photosynthetic efficiency and therefore CO_2_ assimilation rates. Another major difference concerns the growing conditions. Basil plants were cultivated hydroponically under a 3:7 blue-to-red light spectrum at an intensity of 150 µmol m^-^² s^-^¹ (Park et al., 2016), whereas outdoor cultivation without an adjustable light source was applied under natural conditions (Coutinho et al., 2022). Numerous studies on basil have shown that the CO_2_ assimilation rate depends on both light quantity (e.g., increased DLI (Dou et al., 2018; Solis-Toapanta and Gómez, 2019) and light quality (Lin et al., 2021). In addition to light quality and quantity, other abiotic factors affect photosynthesis in the long term. These include growth temperature (Barickman et al., 2021), water and nutrient balance (Kalamartzis et al., 2020; Germano et al., 2022).

Furthermore, no differences were observed between an individual measurement at high irradiances and a light curve with a gradual increase in intensity in either the light reaction or the Calvin cycle. Contrary to expectations, there was no shock reaction with reduced photosynthetic performance. These observations may indicate that central regulatory mechanisms of photosynthesis are not restricted to activation at low irradiance.

### 4.2 Age related shifts in photosynthetic performance

Flowers function as major resource sinks, as the formation of flowers and seeds is a resource-intensive process. This process involves the reallocation of energy, nitrogen, phosphate and other nutrients to flowers (Chapin et al., 1990). While most of a plant’s nitrogen is found in photosynthetic enzymes (e.g., RuBisCO), it is possible that these nutrients are reallocated to flower formation (Millard, 1988). At the same time, the light reaction continuously supplies the necessary energy for this process. However, this means that these resources are no longer available for primary metabolism, such as CO_2_ assimilation, which is consequently reduced (Wardlaw, 1990; Pereira, 1994). It has also been observed that inflorescence has a significant negative effect on the CO_2_ assimilation rate in grapevines (Sawicki et al., 2017) and mangoes (Urban et al., 2008).

Several mechanisms may explain the increase in photosynthetic efficiency between the third and seventh week. To maintain CO_2_ assimilation despite reduced stomatal gas exchange, mesophyll conductance in the leaf may have increased. Mesophyll conductance refers to the mesophyll’s ability to allow CO_2_ to diffuse to the chloroplasts. An increase in mesophyll conductance would prevent a CO_2_ bottleneck caused by lower stomatal conductance (Flexas et al., 2008). It has been established that intercellular airspaces increase in size as a leaf develops. The first air spaces form at an early stage, while the stomatal pattern is simultaneously formed in the epidermis. The subsequent coordination of the air spaces with the stomatal openings only occurs in subsequent stages of development (Baillie and Fleming, 2020), which may explain the observed pattern. Increased mesophyll conductance can improve water use efficiency under conditions of limited water availability, as less stomatal gas exchange is required, which in turn is reflected in a reduced transpiration rate.

### 4.3 Development of sun and shade leaf characteristics along the vertical canopy gradient

Morphological adaptations are unlikely to explain the observed decline in CO_2_ assimilation from the upper to the lower leaf layers due to the age-related phenotypic plasticity of leaves (Fritz et al., 2018). For instance, during cultivation, a leaf responds to increased irradiance by enlarging its palisade parenchyma, thereby enabling higher photosynthetic performance (Yano and Terashima, 2004). However, this plasticity decreases as the leaf ages. The greatest morphological change is only possible within a narrow developmental window, during which the leaf’s structure is characterized by increased cell division and differentiation. Once leaves have passed this juvenile stage, with cell development and leaf structuring largely complete, they can only respond to new stimuli to a limited extent (Sims and Pearcy, 1992; Niinemets, 2016). As many proteins of the light reactions are tightly associated with thylakoid membranes, whereas Calvin cycle enzymes are soluble in the stroma, adjustments in photosynthetic activity are more likely driven by changes in the abundance and regulation of Calvin cycle proteins. A large number of studies support the assumption that RuBisCO concentration or activity may be a decisive factor in photosynthetic adaptation. In rice, wheat, and millet it was shown that, after reaching a maximum, RuBisCO concentration decreases continuously with age across species (Irving and Robinson, 2006). Furthermore, other studies have shown that RuBisCO concentration and activity both decrease with increasing leaf age in, rice and wheat (Suzuki et al., 1987; Xiong et al., 2025). Notably, all studies only considered the age of the leaf, rather than its position on the plant. These studies focused on leaves exposed to maximum irradiance during the initial stages of development, which were subsequently overshadowed by other leaves or entire leaf layers as they aged. Consequently, the light supply also changed. It is well known that RuBisCO and its activase are activated by light (Lan et al., 1992; Jensen, 2004). Assuming that the CO_2_ assimilation rate is directly related to RuBisCO concentration and activity, the question arises as to whether the age of the leaf or the reduced light supply explains the significant differences in this rate. To address this question, horizontally grown tomatoes were studied, ensuring that all leaves were exposed to the same irradiance. The study revealed that the CO_2_ assimilation rate of old leaves was similar to that of young leaves. Consequently, in vertically growing tomatoes, the CO_2_ assimilation rate is reduced by shading caused by leaf layers rather than by leaf age (Trouwborst et al., 2011).

### 4.4 Leaf-side-specific chlorophyll fluorescence

Different illumination and measurement configurations resulted in distinct chlorophyll fluorescence responses between the upper and lower leaf sides. This leaf-side-specific fluorescence has already been documented in earlier studies. In woody plants such as linden or aspen (Mänd et al., 2013), and in herbaceous plants such as rice (Kumagai et al., 2014) and sunflowers (Zou et al., 2022), a higher quantum yield was found on the upper side of the leaf than on the lower side. In eucalyptus and pepper, it has also been demonstrated that the quantum yield decreases with increasing leaf depth, beginning at the upper leaf surface (Evans et al., 1993; Terashima et al., 2009). This leaf-side-specific fluorescence is most likely explained by the anatomical structure of the leaf. The upper side of the leaf has denser palisade tissue with a higher chloroplast density, while the underside consists of loose spongy parenchyma that primarily serves to optimize gas exchange and contains fewer chloroplasts. This results in a vertical light absorption gradient in the leaf, which is reflected by a vertical gradient of fluorescence parameters (Evans et al., 1993; Lichtenthaler et al., 2005).

### 4.5 Neglected wavelengths: the potential of transitional regions between blue, green and red

In addition to studying wavelengths in the green gap, we examined alternative blue and red color tones. Although comparing the absolute values of studies with different cultivation and experimental conditions is difficult, comparing the absolute values with Jokic’s 2025 work is ideal due to the identical cultivation strategy and experimental setup. In that study, conventional blue and red light with wavelengths of 450 and 660 nm, with maximal CO_2_ assimilation rates of 8.9 and 11.2 µmol m^-^² s^-^¹, respectively, were observed (Jokic et al., 2025). Surprisingly, a comparison to this study shows that shifting from 450 to 430 nm increases the CO_2_ assimilation rate by 35%. This raises the question of whether shifting the wavelength by 20 nm increases the absorption capacity of blue light enough to promote photosynthesis or if the shift merely reduces the increased production of secondary metabolites observed in the study. While chlorophyll b exhibits a strong absorption peak around 450 nm, chlorophyll a absorbs most efficiently at slightly shorter wavelengths, with a maximum near 430 nm (Lichtenthaler and Buschmann, 2001). Due to the typically high ratio of chlorophyll a to b in C_3_ plants (Taiz et al., 2015), this shift in wavelength may enhance the overall absorption of light by the photosystems, thereby contributing to the observed increase in CO_2_ assimilation. However, if increased photosynthesis translates into enhanced growth rate, then varying blue light appears to offer a promising agricultural application. Specifically modifying blue wavelengths (430/450 nm) could differentiate between increased plant growth resulting from increased CO_2_ fixation and the accumulation of bioactive and health-promoting substances. Given these findings, cultivation experiments that consider these aspects appear to be a promising approach for further investigation. While the response to the shift in the blue wavelength range is pronounced, only limited photosynthetic sensitivity was observed in the red spectral range. Reducing the wavelength from 660 nm to 590/625 nm leads to a minimal 2% change in the CO_2_ assimilation rate.

Notably, the wavelength range between 485 and 500 nm has not yet been investigated at irradiances above 200 µmol m^-^² s^-^¹, but induces the highest CO_2_ assimilation. Combining the spectral ranges of blue and green light probably utilizes the advantageous absorption properties of both colors, explaining the high photosynthetic performance. The blue spectral range has high absorption compared to green light, and, on the other hand, the green spectral range allows good light penetration (Lichtenthaler and Buschmann, 2001). This likely results in efficient light absorption combined with more homogeneous internal leaf illumination. Another property of blue-green wavelengths is their high photon energy. Increased light absorption, improved penetration of leaf tissue, and high energy discharge cause leaves to heat up significantly. In addition to the increased demand for CO_2_, high transpiration cooling is also required, which explains the increased transpiration rate and stomatal conductance values.

For several reasons, comparing this study to existing literature is ineffective. First, the wavelengths investigated are not commonly studied in current research, except in the context of the action spectrum. In this context, irradiances of up to 200 µmol m^-^² s^-^¹ were only achieved (McCree, 1971; Inada, 1976; Gao et al., 2024). The data obtained in this study clearly show that differences between individual wavelengths increase with rising irradiance up to the light saturation point. The relative proportion of these differences nearly doubles from 500 µmol m^-^² s^-^¹ to 1500 µmol m^-^² s^-^¹. The spectra recorded by McCree were based on wavelengths with an intensity less than half that of the 500 µmol m^-^² s^-^¹ used here. Wavelength-specific effects, such as reduced absorption, improved green light penetration, and better photoprotective properties, only occur at higher irradiances and are not represented by the existing action spectrum.

### 4.6 Enhancing white-light spectra for improved photosynthetic performance

Supplementing neutral white light with two different red tones (660 nm and 625 nm) in three different mixing ratios did not increase photosynthesis. Adding 25% cyan or green light, or 25% or 50% blue light, however, led to a significant increase in photosynthesis. However, increasing the proportion further causes the CO_2_ assimilation rate to return to that of the specific monochromatic light. Therefore, white light supplemented with 25% blue or green light is an almost optimal, balanced spectral mixture. Increasing the red-light proportion reduces both the transpiration rate and stomatal conductance. Low gas exchange also reduces CO_2_ assimilation. An increase in the blue or green proportion, on the other hand, leads to stomatal opening. However, if the proportion of blue light increases too much, the leaf temperature also rises. This indicates that blue light acts as a stress factor and that energy is invested in photoprotective processes, which reduces the CO_2_ assimilation rate (El-Esawi et al., 2017; Jokic et al., 2025). Excessive proportions of green, on the other hand, leads to lower absorption and photosynthetic performance. A similar situation occurs with an increased red or blue component. These wavelengths predominantly illuminate the upper cell layers while insufficiently stimulating deeper tissue (Cui et al., 1991). Balanced, cool white light, however, covers a significant part of the spectrum between the blue and red ranges and effectively stimulates upper as well as deeper cell layers. This spectral composition promotes more homogeneous light absorption and uniform photosynthesis across the entire leaf. Unilateral increases in wavelength disrupt this balance and hinder photosynthesis.

## 5 Conclusion

In addition to age-, leaf-layer-, and leaf-side-specific photosynthesis, it was demonstrated that wavelengths within the green gap range, largely unaddressed at high irradiance, maximize photosynthesis/CO_2_ assimilation among monochromatic wavelengths. At low irradiances, differences between wavelengths were negligible, whereas at higher irradiances approaching light saturation, these differences became increasingly pronounced. At these irradiances, the photoprotective advantages of lower absorption and more homogeneous leaf illumination by greenish wavelengths become apparent. Although leaf absorptance was not determined in this study, the high CO_2_ assimilation rates observed for wavelengths within the green gap do not challenge McCree’s classical action spectrum but may highlight potential limitations under high irradiance conditions. Future studies should combine gas exchange and spectral absorptance measurements across different species at light intensities close to the light saturation point to establish a potential irradiance dependent action spectrum. Additionally, optimal balanced white-light spectra were identified as promising for future growth experiments due to the highest CO_2_ assimilation rates observed. The largely untapped potential of cyan/mint green light is also promising in this regard.

## Data Availability

The raw data supporting the conclusions of this article will be made available by the authors, without undue reservation.
