# Supplementary material for: Photosynthesis in transition: the importance of wavelengths in the green gap for Ocimum basilicum L
Source: Front Plant Sci. 2026 Jun 19;17:1842244. doi: 10.3389/fpls.2026.1842244 (PMC13328402; doi:10.3389/fpls.2026.1842244)
Supplement: Supplementary file 1 [file DataSheet1.docx]

Supplementary Material

# Supplementary Figures and Tables

Supplementary Table 1: Comparison of CO₂ assimilation rate [A] in µmol m⁻² s⁻¹ at different irradiances in µmol m⁻² s⁻¹ (4000 K), obtained through a light curve measurement procedure and individual measurements (n = 10 per light curve and per irradiance of individual measurements, corresponding to Fig. 4A). Statistical significance was determined by one-way ANOVA (p ≤ 0.05). Different lowercase letters indicate statistically significant differences among treatments within the same irradiance level

| Light program /irradiance | 0 µmol m⁻² s⁻¹ | 500 µmol m⁻² s⁻¹ | 1500 µmol m⁻² s⁻¹ | 2500 µmol m⁻² s⁻¹ | 3500 µmol m⁻² s⁻¹ | 5000 µmol m⁻² s⁻¹ |
| --- | --- | --- | --- | --- | --- | --- |
| A [µmol m⁻² s⁻¹] Lightcurve | -0,26 ± 0 ^a^ | 9,05 ± 0,86 ^a^ | 11,90 ± 1,14 ^a^ | 12,05 ± 0,75 ^a^ | 12,37 ± 0,64 ^a^ | 12,62 ± 0,99 ^a^ |
| A [µmol m⁻² s⁻¹] Individual measurement | -0,26 ± 0 ^a^ | 8,89 ± 0,8 ^a^ | 11,73 ± 1,03 ^a^ | 12,03 ± 0,99 ^a^ | 12,26 ± 1 ^a^ | 11,66 ± 1 ^a^ |

Supplementary Table 2: Comparison of quantum yield of photosystem II [Y(II)] at different irradiances in µmol m⁻² s⁻¹ (2600 K), obtained through a light curve measurement procedure and individual measurements (n = 10 per light curve and per irradiance of individual measurements, corresponding to Fig. 4B). Statistical significance was determined by one-way ANOVA (p ≤ 0.05). Different lowercase letters indicate statistically significant differences among treatments within the same irradiance level

| Light program /irradiance | 0 µmol m⁻² s⁻¹ | 285 µmol m⁻² s⁻¹ | 600 µmol m⁻² s⁻¹ | 1100 µmol m⁻² s⁻¹ | 1800 µmol m⁻² s⁻¹ | 2500 µmol m⁻² s⁻¹ | 3500 µmol m⁻² s⁻¹ | 4800 µmol m⁻² s⁻¹ | 5800 µmol m⁻² s⁻¹ | 6900 µmol m⁻² s⁻¹ | 7300 µmol m⁻² s⁻¹ |
| --- | --- | --- | --- | --- | --- | --- | --- | --- | --- | --- | --- |
| Y(II) Lightcurve | 0,79 ± 0 ^a^ | 0,66 ± 0,02 ^a^ | 0,51 ± 0,05 ^a^ | 0,40 ± 0,04 ^a^ | 0,29 ± 0,03 ^a^ | 0,26 ± 0,02 ^a^ | 0,21 ± 0,03 ^a^ | 0,18 ± 0,03 ^a^ | 0,17 ± 0,03 ^a^ | 0,16 ± 0,03 ^a^ | 0,16 ± 0,03 |
| Y(II) Individual measurement | 0,79 ± 0,03 ^a^ | 0,71 ± 0,04 ^a^ | 0,46 ± 0,03 ^a^ | 0,38 ± 0,03 ^a^ | 0,27 ± 0,04 ^a^ | 0,25 ± 0,02 ^a^ | 0,22 ± 0,03 ^a^ | 0,19 ± 0,02 ^a^ | 0,17 ± 0,04 ^a^ | 0,17 ± 0,04 ^a^ | - |

Supplementary Table 3: Comparison of quantum yield of PS II [Y(II)] at different irradiances in µmol m⁻² s⁻¹ between the different experimental approaches to measure chlorophyll fluorescence (n = 10 per measurement condition, corresponding to Fig. 6). Statistical significance was determined by one-way ANOVA (p ≤ 0.05). Different lowercase letters indicate statistically significant differences among treatments within the same irradiance level

| Measurement condition /irradiance | 0 µmol m⁻² s⁻¹ | 285 µmol m⁻² s⁻¹ | 500 µmol m⁻² s⁻¹ | 600 µmol m⁻² s⁻¹ | 1100 µmol m⁻² s⁻¹ | 1500 µmol m⁻² s⁻¹ | 1800 µmol m⁻² s⁻¹ | 2500 µmol m⁻² s⁻¹ |
| --- | --- | --- | --- | --- | --- | --- | --- | --- |
| Y(II) adaxial illuminated and measured | 0,78 ± 0 ^a^ |  | 0,67 ± 0,02 ^a^ |  |  | 0,59 ± 0,03 ^a^ |  | 0,54 ± 0,03 ^a^ |
| Y(II) abaxial illuminated and adaxial measured | 0,79± 0 ^a^ |  | 0,63 ± 0,03 ^a^ |  |  | 0,45 ± 0,04 ^b^ |  | 0,38 ± 0,04 ^b^ |
| Y(II) adaxial illuminated and abaxial measured | 0,79 ± 0 ^a^ | 0,66 ± 0,02 |  | 0,51 ± 0,05 | 0,40 ± 0,04 |  | 0,29 ± 0,03 | 0,26 ± 0,02 ^c^ |

Supplementary Table 4: Comparison of transpiration rate [E] in mmol m⁻² s⁻¹ at different irradiances in µmol m⁻² s⁻¹, with different light colors (neutral white: 4000 K, deep blue: 430 nm, cyan: 485 nm, mint green: 500 nm, orange: 590 nm, and orange-red: 625 nm, n = 10 per light color, corresponding to Fig. 8A). Statistical significance was determined by one-way ANOVA (p ≤ 0.05). Different lowercase letters indicate statistically significant differences among treatments within the same irradiance level

| Wavelength /irradiance | 0 µmol m⁻² s⁻¹ | 500 µmol m⁻² s⁻¹ | 1500 µmol m⁻² s⁻¹ | 2500 µmol m⁻² s⁻¹ | 3500 µmol m⁻² s⁻¹ | 5000 µmol m⁻² s⁻¹ |
| --- | --- | --- | --- | --- | --- | --- |
| E [mmol m⁻² s⁻¹] at 4000 K | 0,66 ± 0 ^a^ | 1,96 ± 0,2 ^a^ | 2,63 ± 0,25 ^a^ | 3,04 ± 0,21 ^a^ | 3,53 ± 0,23 ^a^ | 4,15 ± 0,31 ^a^ |
| E [mmol m⁻² s⁻¹] at 430 nm | 0,66 ± 0 ^a^ | 2,16 ± 0,18 ^a,c^ | 3,04 ± 0,22 ^c,d^ | 3,93 ± 0,25 ^c^ | 4,77 ± 0,33 ^c^ | 5,8 ± 0,47 ^c^ |
| E [mmol m⁻² s⁻¹] at 485 nm | 0,66 ± 0 ^a^ | 2,32 ± 0,14 ^c^ | 3,27 ± 0,20 ^c^ | 4,12 ± 0,18 ^c^ | 4,91 ± 0,22 ^c^ | 5,98 ± 0,27 ^c^ |
| E [mmol m⁻² s⁻¹] at 500 nm | 0,66 ± 0 ^a^ | 1,89 ± 0,25 ^a,b^ | 2,82 ± 0,32 ^a,d^ | 3,82 ± 0,39 ^c^ | 4,65 ± 0,45 ^c^ | 5,48 ± 0,66 ^c^ |
| E [mmol m⁻² s⁻¹] at 590 nm | 0,66 ± 0 ^a^ | 1,61 ± 0,14 ^b,c^ | 2,07 ± 0,17 ^b^ | 2,49 ± 0,22 ^b^ | 2,90 ± 0,24 ^b^ | 3,66 ± 0,35 ^b^ |
| E [mmol m⁻² s⁻¹] at 625 nm | 0,66 ± 0 ^a^ | 1,77 ± 0,19 ^a,b^ | 2,28 ± 0,22 ^a,b^ | 2,67 ± 0,24 ^b^ | 3,11 ± 0,24 ^b^ | 3,73 ± 0,35 ^a,b^ |

Supplementary Table 5: Comparison of leaf temperature [T] in °C at different irradiances in µmol m⁻² s⁻¹, with different light colors (neutral white: 4000 K, deep blue: 430 nm, cyan: 485 nm, mint green: 500 nm, orange: 590 nm, and orange-red: 625 nm, n = 10 per light color, corresponding to Fig. 8B). Statistical significance was determined by one-way ANOVA (p ≤ 0.05). Different lowercase letters indicate statistically significant differences among treatments within the same irradiance level

| Wavelength /irradiance | 0 µmol m⁻² s⁻¹ | 500 µmol m⁻² s⁻¹ | 1500 µmol m⁻² s⁻¹ | 2500 µmol m⁻² s⁻¹ | 3500 µmol m⁻² s⁻¹ | 5000 µmol m⁻² s⁻¹ |
| --- | --- | --- | --- | --- | --- | --- |
| T [°C] at 4000 K | 24,45 ± 0 ^a^ | 24,47 ± 0,07 ^a^ | 27,78 ± 0,18 ^a^ | 25 ± 0,15 ^a^ | 25,40 ± 0,29 ^a^ | 25,91 ± 0,5 ^a^ |
| T [°C] at 430 nm | 24,45 ± 0 ^a^ | 24,78 ± 0,12 ^b^ | 25,91 ± 0,42 ^b^ | 26,62 ± 0,62 ^c,b^ | 27,79 ± 0,9 ^c^ | 29,26 ± 1,3 ^c^ |
| T [°C] at 485 nm | 24,45 ± 0 ^a^ | 24,63 ± 0,13 ^a,b^ | 26 ± 0,34 ^b^ | 27,41 ± 0,25 ^c,d^ | 29 ± 0,39 ^c^ | 30,78 ± 0,54 ^c^ |
| T [°C] at 500 nm | 24,45 ± 0 ^a^ | 24,73 ± 0,14 ^b^ | 26,12 ± 0,18 ^b^ | 27,66 ± 0,23 ^d^ | 29,02 ± 0,32 ^c^ | 30,14 ± 0,67 ^c^ |
| T [°C] at 590 nm | 24,45 ± 0 ^a^ | 24,53 ± 0,08 ^a^ | 25,12 ± 0,16 ^a^ | 25,86 ± 0,35 ^b^ | 26,48 ± 0,51 ^b^ | 27,36 ± 0,72 ^b^ |
| T [°C] at 625 nm | 24,45 ± 0 ^a^ | 24,52 ± 0,07 ^a^ | 25,06 ± 0,3 ^a^ | 25,60 ± 0,54 ^a,b^ | 26,10 ± 0,75 ^a,b^ | 26,92 ± 1,13 ^a,b^ |


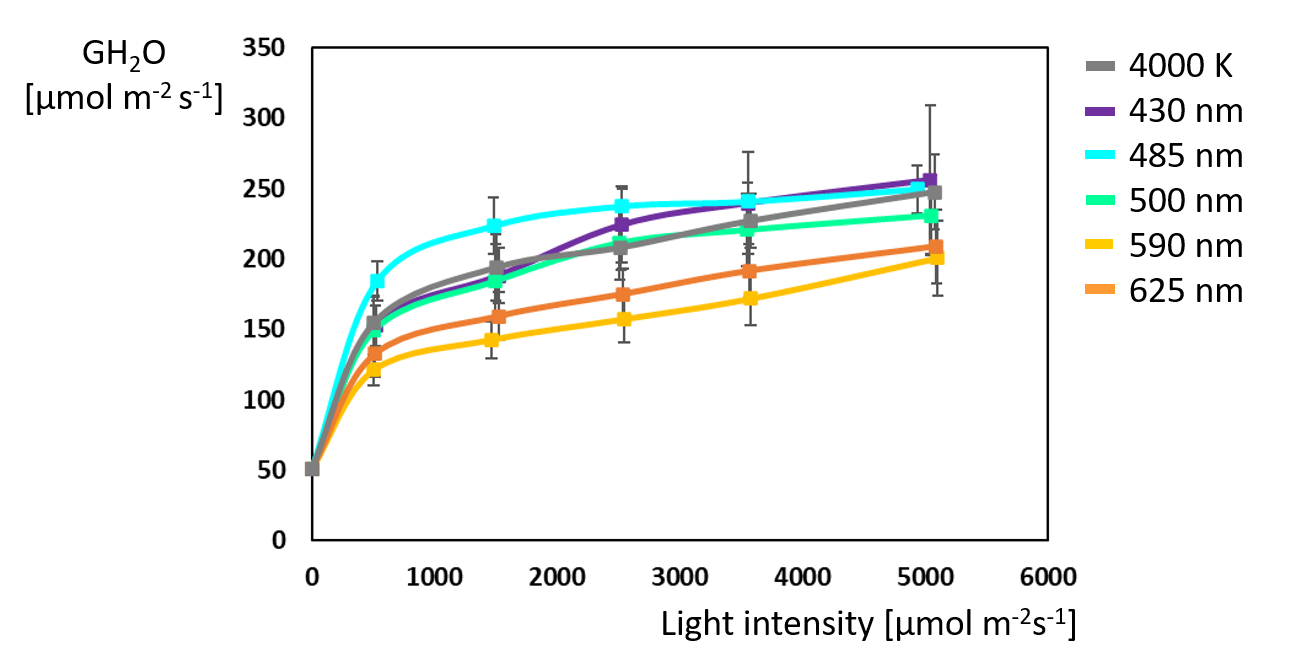

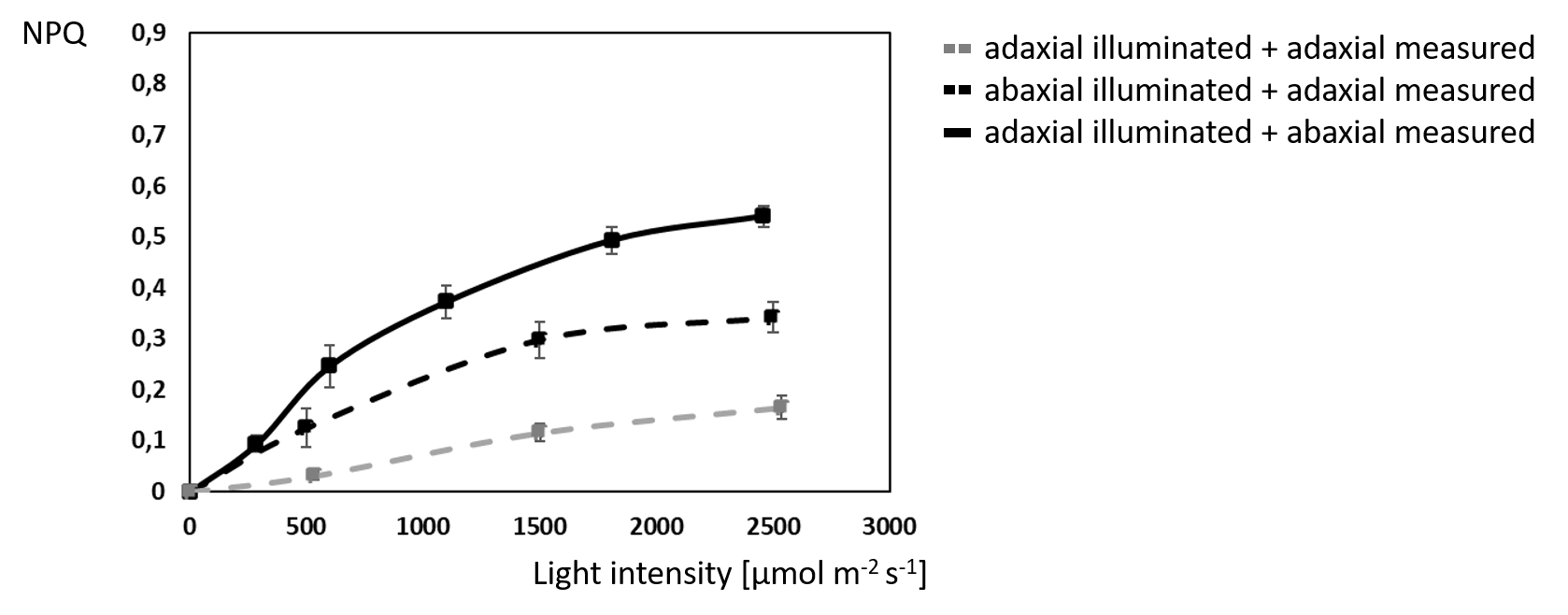


Supplementary Figure 1: Comparison of the non-photochemical quenching [NPQ] as a function of irradiance in µmol m⁻² s⁻¹ between the different experimental approaches to measuring chlorophyll fluorescence (n = 10 per measurement condition)

**Supplementary Figure 2.** Comparison of the stomatal conductance [GH_2_O] in µmol m⁻² s⁻¹ as a function of irradiance in µmol m⁻² s⁻¹, under different light colors (neutral white: 4000 K, deep blue: 430 nm, cyan: 485 nm, mint green: 500 nm, orange: 590 nm, and orange-red: 625 nm, n = 10 per light color)


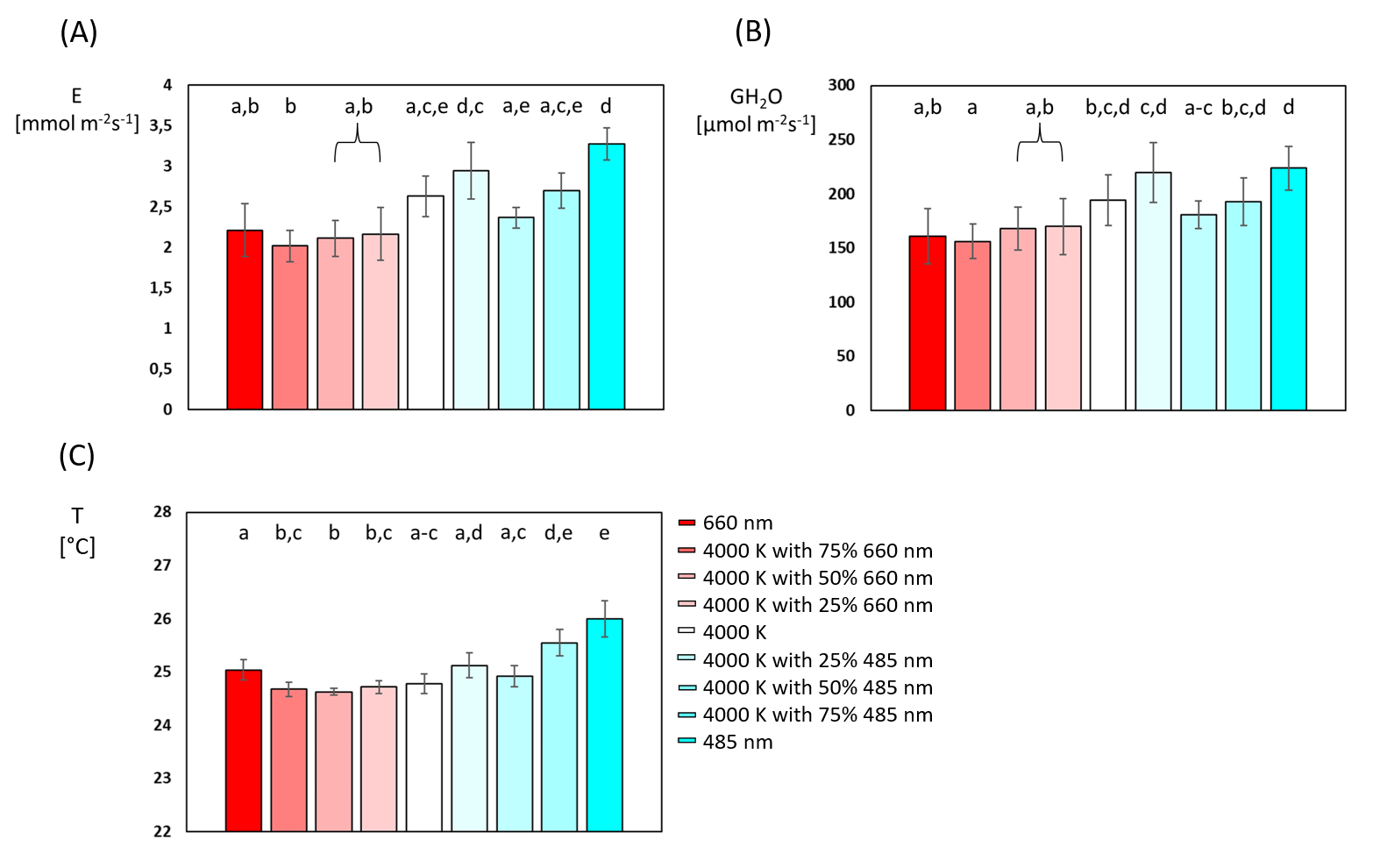


**Supplementary Figure 3.** Comparison of (A) transpiration rate [E] in mmol m⁻² s⁻¹, (B) stomatal conductance [GH_2_O] in µmol m⁻² s⁻¹ and (C) leaf temperature [T] in °C as a function of irradiance in µmol m⁻² s⁻¹, under different white light supplements: neutral white: 4000 K with 0, 25, 50, 75, or 100% red: 660 nm or cyan: 485 nm light, at a irradiance of 1500 µmol m⁻² s⁻¹, (n = 10 per light color)


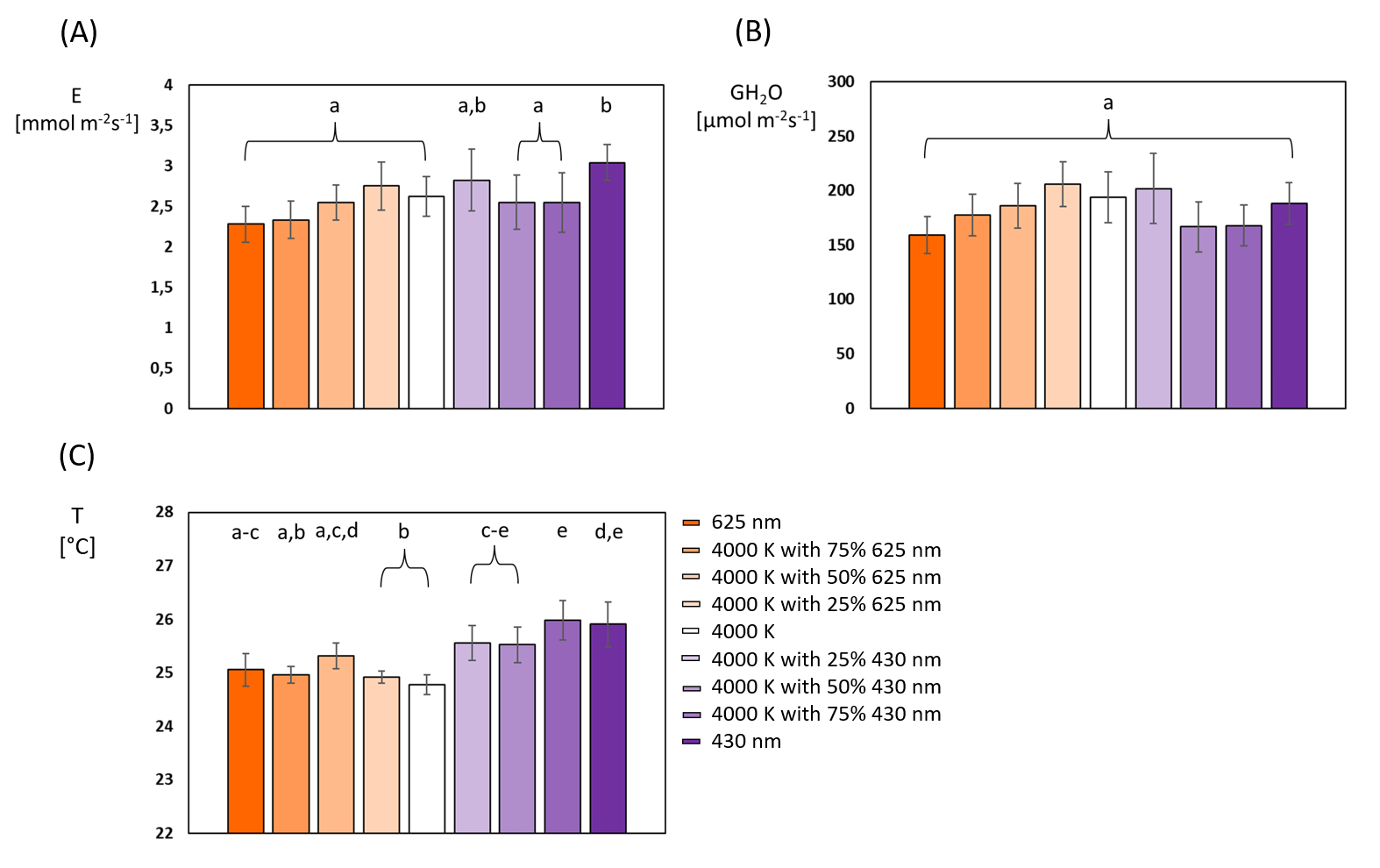


**Supplementary Figure 4.** Comparison of (A) transpiration rate [E] in mmol m⁻² s⁻¹, (B) stomatal conductance [GH_2_O] in µmol m⁻² s⁻¹ and (C) leaf temperature [T] in °C as a function of irradiance in µmol m⁻² s⁻¹, under different white light supplements: neutral white: 4000 K with 0, 25, 50, 75, or 100% orange-red: 625 nm or deep blue: 430 nm light, at a irradiance of 1500 µmol m⁻² s⁻¹, (n = 10 per light color)


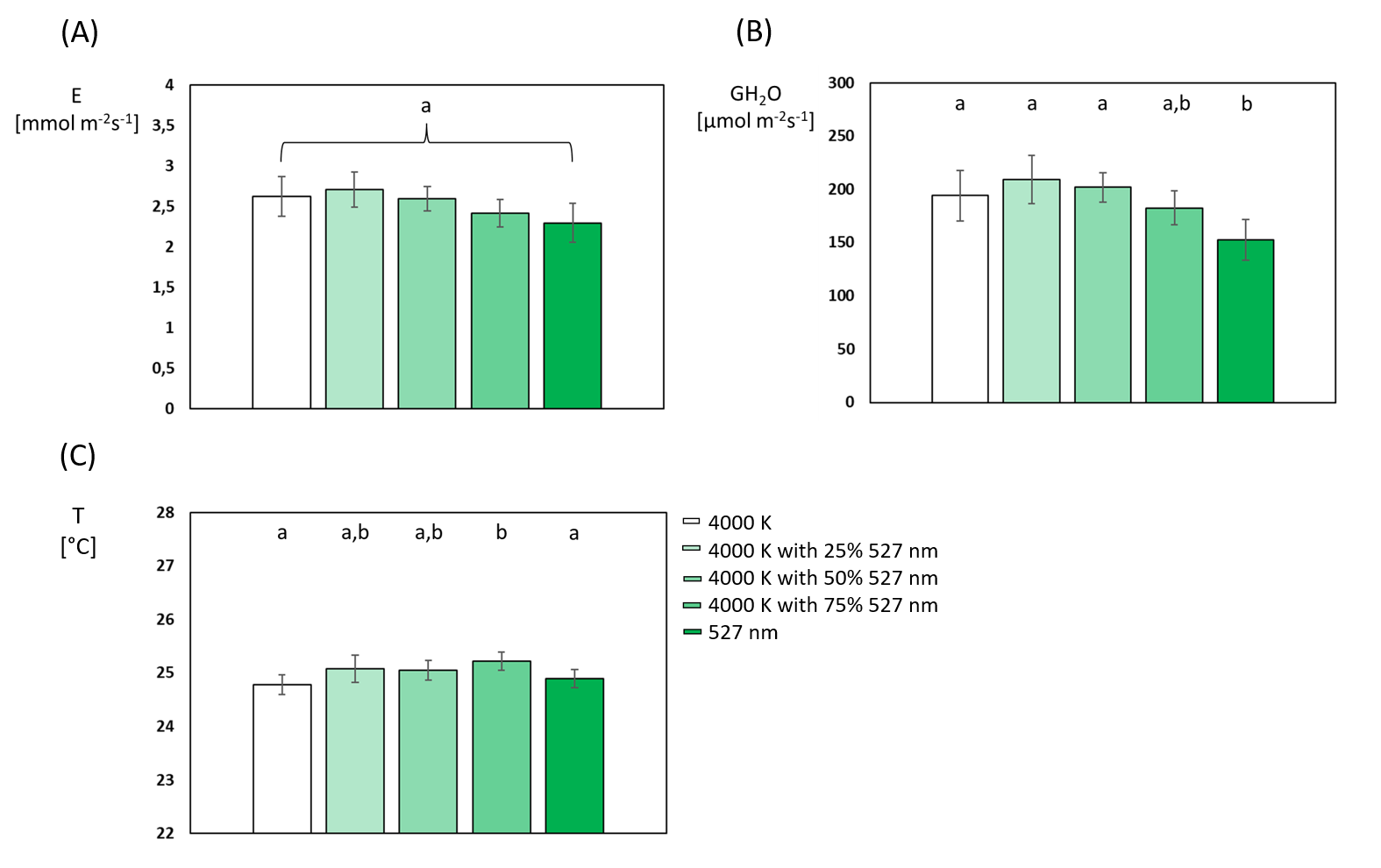


**Supplementary Figure 5.** Comparison of (A) transpiration rate [E] in mmol m⁻² s⁻¹, (B) stomatal conductance [GH_2_O] in µmol m⁻² s⁻¹ and (C) leaf temperature [T] in °C as a function of irradiance in µmol m⁻² s⁻¹, under different white light supplements: neutral white: 4000 K with 0, 25, 50, 75, or 100% green: 527 nm light, at a irradiance of 1500 µmol m⁻² s⁻¹, (n = 10 per light color)
